# A case series associated with different kinds of endo-perio lesions

**DOI:** 10.4317/jced.51219

**Published:** 2014-02-01

**Authors:** Hacer Aksel, Ahmet Serper

**Affiliations:** 1 Department of Endodontics, Faculty of Dentistry, Hacettepe University, Ankara, Turkey

## Abstract

Pulpal and periodontal problems are responsible for more than half of the tooth mortality. There are some articles published in the literature about this issue. Many of them are quite old. There has been also lack of knowledge about the effect of endodontic treatment on the periodontal tissue healing and suitable treatment interval between endodontic and periodontal treatments. In this case report, different kinds of endo-perio lesion were treated with sequential endodontic and periodontal treatment. The follow-up radiographs showed complete healing of the hard and soft tissue lesions. The tooth with endo-perio lesions should be evaluated thoroughly in terms of any cracks and fracture, especially furcation areas for a long term prognosis. In this case report, it was showed that 3 months treatment intervals between endodontic treatment and periodontal surgery has no harmful effect on periodontal tissue healing.

** Key words:**Endo-perio lesion, furcation, mandibular molar, bone graft, crack line, treatment interval.

## Introduction

Dental pulp and periodontal tissues are closely related that are ectomesenchymal in origin (1). The pulp origina-tes from the dental papilla and the periodontal ligament from the dental follicle and they are separated by Hertwig’s epithelial root sheat.

Pulpal and periodontal problems are responsible for more than half of the tooth mortality (2). The relationship between periodontal and pulpal disease was first described by Simring and Goldberg in 1964 (3). Since then, the term “endo-perio lesion” has been used to describe this type of lesions due to same inflammatory products found in both periodontal and pulpal tissues.

The vast majority of pulpal and periodontal diseases are caused by bacterial infection. It has been suggested that cross-infection between the root canal and the periodontal ligament can occur via the anatomical (apical foramen, lateral and accessory canals, dentinal tubules and palato-gingival grooves) and non-physiological pathways (iatrogenic root canal perforations and vertical root fractures) (4). These pathways determine the spread of infection. Periodontal disease causes destruction of bone in a coronal-to-apical direction while direction of the endodontic lesions is from apex to coronal. When the pulp is infected, it elicits an inflammatory response of periodontal ligament. However, the effect of periodontal inflammation on the pulpal tissue remains controversial (5). Clinically, the pulp is not affected by periodontal disease until accessory canals are exposed to the oral environment or microvasculature of the apical foramen is damaged (6).

The classification of endo-perio lesions by Simon et al. is that primary endodontic diseases, primary periodontal diseases and combined disease including primary endodontic disease with secondary periodontal involvement, primary periodontal disease with secondary endodontic involvement and true combined disease (7). This classification has been used and given very valuable guidance to make sound clinical decisions.

The main factors to take into account for treatment decision-making are pulp vitality and type and extent of periodontal defect. The differential diagnosis of endodontic and periodontal diseases can be challenged but a correct diagnosis has a vital importance so that appropriate treatment can be provided.

The aim of this study was to present the diagnosis and management of different types endo-perio lesions and emphasize the importance of the correct treatment sequence.

## Primary Endodontic Disease

- Case 1

**Figure 1:**
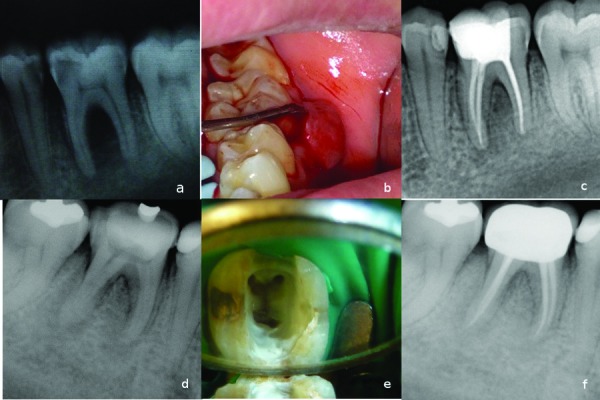
Case 1- a) Initial radiograph of tooth #36. b) Intraoral film showing increased probing depth values in the furcation area. c) A year recall radiograph showing complete healing. Case 2- d) Initial radiograph showing increased bone lesion in the furcation and distal side of the tooth #46. e) Access opening showing mesio-distal crack line in the pulp floor. f) A year recall radiograph showing healing of the bone lesion.

A 21-year-old male patient, with a noncontributory medical history was referred for the treatment of pain and intraoral localized swelling in the left mandibular first molar. Clinical and radiographic examinations revealed large caries and periapical and furcal lesions related to tooth #36 (Fig. 1). There was a localized swelling on the gingival sulcus. The tooth mobility was grade II and periodontal probing through the furcation showed increased probing values with grade II defect (Fig. 1). An electric pulp test (Parkell Electronics Division, Farmingdale, NY) displayed negative response. Endodontic treatment was administered in two visits, with an interappointment calcium hydroxide medication. A week later, the localized swelling was quite resolved, the symptoms of the tooth was disappeared and the root canal treatment was completed. No periodontal treatment was rendered. A year follow-up radiograph showed complete soft and hard tissue repairs in the periapical and furcal area of tooth #36 (Fig. 1).

- Case 2

A 45-year-old female patient, whose medical history was noncontributory, came to our department for evaluation and treatment of tooth #46. She complained discomfort on chewing, related to tooth #46. Clinical and radiographic examination revealed a sinus tract and radiolucent lesion in the furcal and distal side of tooth #46 (Fig. 1).

Periodontal probings were increased at the furcal and distal side of tooth #46. The tooth gave negative response to vitality tests. After endodontic access cavity was established, a mesio-distal crack line was observed (Fig. 1). Root canal treatment completed and occlusal reduction was performed. The patient was referred for prosthetic crown. After a year following period, the repair of lesion was observed and the tooth was asymptomatic (Fig. 1).

## Primary periodontal disease with secondary endodontic involvement

- Case 3

**Figure 2:**
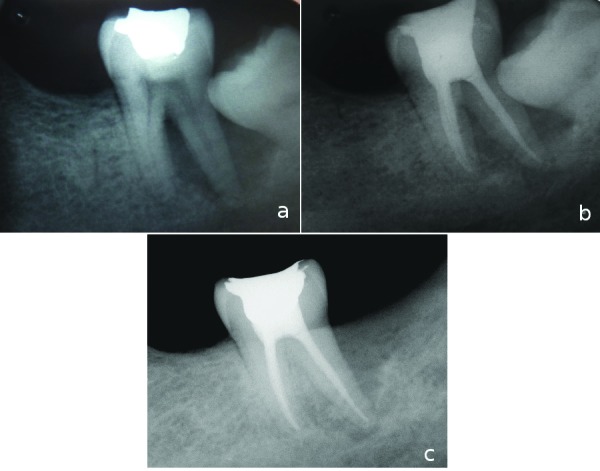
a) Initial radiograph of tooth #37. b) Final radiograph after obturation of the root canals. c) A year recall radiograph showing complete healing of the bone lesion.

A 42-year-old male patient, whose medical history was noncontributory, came to our department with a history of acute pain and localized swelling in the left mandibular molar area. Radiographic examination presented severe bone loss around the distal root of tooth #37 (Fig. 2). The cause of the bone loss was considered related to the uncleaning space between second and third molar. The rest of the dentition had normal periodontal values. The tooth #37 was non responsive to vitality tests. After completion of root canal treatment, the patient was referred for extraction of the third molar tooth (Fig. 2). At the 6 month recall visit, the complete repair of the bony lesion was observed (Fig. 2).

## Primary endodontic disease with secondary periodontal involvement

- Case 4

A 45-year-old woman presented to inquire about options for preserving tooth #36. The patient’s medical status was noncontributory. The tooth was characterized by gingival reddening and swelling at the buccal side. The patient complained of periodic discharge of pus from the periodontal pocket, sensitivity on percussion, tooth mobility, and intermittent pain.

**Figure 3:**
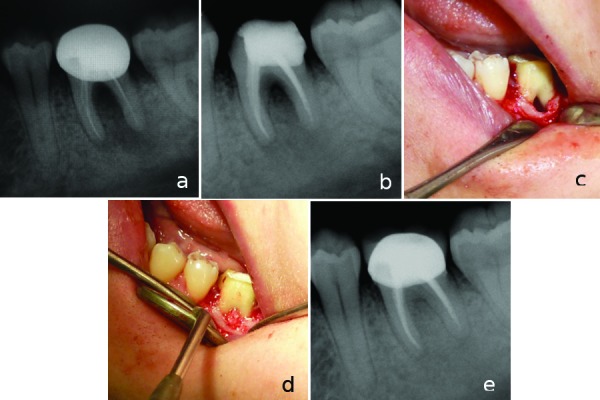
a) Initial radiograph of tooth #36. b) 3 months recall radiograph showing bone lesion in the furcation area. c) Intraoral film showing furcation lesion during periodontal surgery. d) Intraoral film showing bone graft material placement to the furcation lesion. e) Two year recall radiograph showing complete healing of the furcation lesion.

Radiographs displayed a bony defect in the furcal and periapical area of tooth #36, which had unsuccessful root canal treatment (Fig. 3). The probing depth in the furcal area was 12 mm and probable throughout (grade III furcal lesion). The patient informed about the methods and risks of the treatment. Endodontic pretreatment was performed and treatment results were evaluated 3 months later which showed that the furcation lesion still remained intact (Fig. 3). Therefore, periodontal regenerative surgery was planned for the treatment of the furcation defect.

After administering of local anesthesia, a mucoperiosteal flap was raised at the buccal aspect, following intracrevicular incisions and vertical releasing incision. A vertical releasing incision was placed, extending into the alveolar mucosa not closer than one tooth to the involved area. After reflection thorough degranulation and debridement was done at the defect area. Also thorough scaling and root planning was carried out on the exposed root surface area of the defect. After instrumentation, the root surfaces were washed with saline solution to attempt to remove any remaining detached fragments from the defect and surgical field. After that, the bone defect was filled with a xenograft material (Osteobiol GenOs, Tecnoss) and stabilized in the furcation area (Fig. 3). Primary soft tissue closure of the flap was done with nonresorbable black silk [3-0] suture (Ethicon, Inc. Somerville, NJ) using interrupted suturing technique. The patient was advised proper plaque control, and prescribed 0.12% chlorhexidine mouthwash for rinsing twice daily, for a week. The sutures were removed 10 days after surgery. A year recall radiograph showed complete bone repair in the furcation defect (Fig. 3).

## Discussion

The endo-perio lesions present challenges to clinicians as far as diagnosis and prognosis of the involved teeth are concerned. Correct diagnosis is essential prerequisite to determine the treatment and long-term prognosis. Diagnosis of primary endodontic disease and primary periodontal disease usually presents no clinical difficulty. The first step for proper diagnosis is the vitality tests. Although the vitality test cannot provide the histological status of the dental pulp, their ability to register pulp vitality is quite satisfied. The ability of vitality tests to detect non-sensitive reaction represented a necrotic pulp was reported as 89% with the cold test and 88% with the elec-trical test (8).

The infected root canal can cause a chronic inflammatory reaction which extends the gingival sulcus and drains through the sinus tracts. If the rest of the dentition is periodontally healthy and any root cracks and fractures has been ruled out, healing of the periodontal tissues can be expected after endodontic treatment as it was observed in case 1 and 2. Therefore, further treatment requirements should always considered followed by an observation period of at least 3 months. Conversely, there has been a debate in the literature about the impact of the endodontic treatment on the healing potential of the periodontium. Some studies have been reported that endodontic treatment may cause an inhibitory effect on periodontal wound healing (9,10) while some of them (11,12) have been demonstrated no significant effects. The possible influence of endodontic treatment on the healing response of furcation defects is related to the accessory canals and permeable areas of dentin and cementum. Accessory canals in the whole furcation area of molars are found in 30–60% of molars and predispose this area to be a zone of intense communication between pulpal and periodontal tissues (13). These canals are mostly observed in the furcation area of mandibular molars (14).

Proper endodontic treatment is a key factor for treatment success. Poor endodontic treatment allows canal re-infection and in this way, leads to the treatment failure (15). Moreover, there are other contributing factors to cause endo-perio lesions. The tooth should be always evaluated in terms of any artificial pathways between periodontal and pulpal tissues such as cracks and fractures. The source of both infections should be removed. In case 3, the lesion might be caused primarily by periodontal pathogens due to the inaccessible area between mandibular second and third molar teeth . In this process, chronic marginal periodontitis progresses apically along the root surface. Although in most cases, pulp-tests indicate a clinically normal pulpal reaction, there was a pulp necrosis in tooth #37. Because of the fact that the periodontal infection reached to the apical foramen so microvasculature of the apical foramen could be destroyed. The treatment depends on the endodontic treatment of tooth #37 and extraction of the impacted third molar tooth.

In this case report, the cleaning and shaping of the root canals were performed in combination with irrigation with sodium hypochlorite and additional interappointment calcium hydroxide medication to render the root canal system free of cultivable bacteria.

Calcium hydroxide is bactericidal, anti-inflammatory and proteolytic and inhibits resorption and favors repair. It also inhibits periodontal contamination from instrumented canals via patent channels connecting the pulp and periodontium before periodontal treatment removes the contaminants. We observed that the sinus tracts extending into the furcation area were disappeared 1-2 weeks later and the canals are eventually filled with a conventional obturation.

When periodontal and pulpal lesions occur concurrently, it has been described as combined lesion. In this condition, the treatment and prognosis of the tooth are different from those of teeth involved with only primary endodontic disease. The tooth now requires both endodontic and periodontal treatments. If the endodontic treatment is adequate, the prognosis depends on the severity of the periodontal damage and the efficacy of periodontal treatment. If an increased periodontal tissue destruction occur and cannot be replaced with periodontal regenerative techniques, the extraction of the tooth seems the only solution. The bony lesion of case 4 had endodontic and periodontal lesions and first treated with endodontic therapy. Treatment results were evaluated in 2–3 months and then periodontal treatment was considered. This sequence of treatment allows sufficient time for initial tissue healing and better assessment of the periodontal condition. This case demonstrates that proper diagnosis, followed by removal of etiological factors and utilizing the guided tissue regeneration technique combined with osseous grafting, will restore health and function to a tooth with severe attachment loss caused by an endo-perio lesion.

The ideal interval between the endodontic treatment and periodontal surgery has also been challenged by controversial findings. It was reported that root canal treatment performed 2.5 months before periodontal surgery not to impair periodontal healing (11). Miranda et al. suggest that endodontic treatment performed 6 months before the surgical debridement of the furcation of mandibular molars did not impair the clinical parameters of periodontal healing (12). In the present case report, root canal treatment was performed 3-4 months before the periodontal surgery showed no disruptive effect on the complete healing of the furcation lesion of the mandibular molar. This result should be confirmed by future clinical studies.

The treatment planning given in this case report can guide the clinician to deal with the treatment of different types endo-perio lesions. In this case report, treatment strategies were given in related to the different kinds of endo-perio lesions. Treatment outcomes will be more predictable if the clinician has more through knowledge about the diagnosis, treatment sequences and intervals. Thereby, the immediate and true management of the endo-perio lesions can impede the loss of the natural tooth and delay the more complex treatments.
